# MicroRNA-Based Linkage between Aging and Cancer: from Epigenetics View Point

**DOI:** 10.22074/cellj.2016.4303

**Published:** 2016-05-30

**Authors:** Saeid Saeidimehr, Ammar Ebrahimi, Najmaldin Saki, Parisa Goodarzi, Fakher Rahim

**Affiliations:** 1 Naft Grand Hospital, Ahvaz, Iran; 2 Department of Medical Biotechnology, School of Advanced Medical Technology, Tehran University of Medical Sciences, Tehran, Iran; 3 Health Research Institute, Thalassemia and Hemoglobinopathy Research Center, Ahvaz Jundishapur University of Medical Sciences, Ahvaz, Iran; 4 School of Nursing and Midwifery, Iran University of Medical Sciences, Tehran, Iran

**Keywords:** Ageing, Disability, Genomics, Longevity, MicroRNAs

## Abstract

Ageing is a complex process and a broad spectrum of physical, psychological, and social changes over time. Accompanying diseases and disabilities, which can interfere with cancer treatment and recovery, occur in old ages. MicroRNAs (miRNAs) are a set of small non-coding RNAs, which have considerable roles in post-transcriptional regulation at gene expression level. In this review, we attempted to summarize the current knowledge of miRNAs functions in ageing, with mainly focuses on malignancies and all underlying genetic, molecular and epigenetics mechanisms. The evidences indicated the complex and dynamic nature of miRNA-based linkage of ageing and cancer at genomics and epigenomics levels which might be generally crucial for understanding the mechanisms of age-related cancer and ageing. Recently in the field of cancer and ageing, scientists claimed that uric acid can be used to regulate reactive oxygen species (ROS), leading to cancer and ageing prevention; these findings highlight the role of miRNA-based inhibition of the SLC2A9 antioxidant pathway in cancer, as a novel way to kill malignant cells, while a patient is fighting with cancer.

## Introduction

Ageing is a complex process and a broad spectrum of physical, psychological and social changes over time (1). It has been determined as one of the admitted risk factors for most of the human age-related diseases such as cancer, leading to approximately 100,000 people deaths around the world per day (2). Most of the disease and disability, which may interfere with cancer treatment and recovery, occur in old ages. Neoplasm is an undiscerning disease that can affect any part of the body of the human being. Roughly one third of the people are at risk to get cancer in their life (3). However, the incidence of cancer is greatly increased in an age-dependent manner. It is reported that around 60% of all cancers happen in people aged 65 years or above (2). Several molecular mechanisms have linked ageing and cancer together (4). 

Micro-RNAs (miRNAs) are a set of small non-coding RNAs with considerable roles in post-transcriptional regulation at gene expression level. Recent studies revealed that miRNAs are involved in many important biological processes such as proliferation, differentiation, angiogenesis, and immune response. miRNAs are generally divided into two categories: the first category acts as cytoplasm mRNA inhibitory (e.g. miRNA-451, miRNA-31, and miRNA-150) and the second one targets nuclear gene transcription directly (e.g. miR-211) (5-7). Thus far, numerous miRNAs have been reported to be involved in different types of malignancy, such as gastric cancer, highlighting them as potential treatment targets (8,9). 

In this review we attempted to summarize the current knowledge of miRNAs in ageing with mainly focus on malignancies and all underlying genetic, molecular and epigenetic mechanisms. 

## miRNAs and their biogenesis

**Fig.1:**
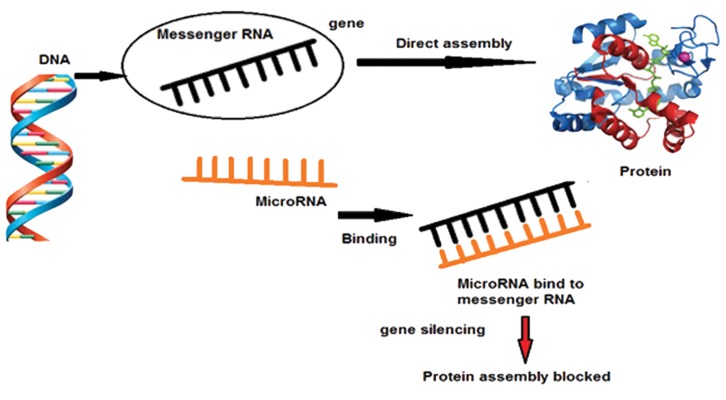
microRNA and inhibiting gene expression.

miRNAs are highly conserved RNA molecules in the cell that regulate gene expression through an interference pathway (10). RNA interference (RNAi) is the post-transcriptional silencing mechanism in eukaryotes that induces degradation of homologous mRNA through creating double stranded RNA (11). miRNAs often bind to the 3'UTR region of the target mRNA, which directs the inhibition of its translation or degradation (12). For example, the product of Lin-4, controlling genes in *Caenorhabditis elegans (C. elegans)*, is a 22 nucleotides RNA that is produced by a 60 nucleotides hairpin precursor, and inhibits translation of Lin14 through interaction with the 3'UTR of this mRNA (10). Distribution of miRNA regions in the human genome is in single or cluster form. Some of these regions, at least half of them, are presented in certain transcription units, such as introns and exons (13). miRNA biogenesis takes place in the nucleus and cytoplasm, while the primary miRNAs, transcribed and polyadenylated by RNA polymerase II, are several kilo-bases (Kbs) (5). Stem-loop structure of this transcript is recognized by a 650 kDa enzyme complex that is presented in the nucleus (14). This complex contains class 2 of the RNase III enzymes, called Drosha, which is specialized to cut a double-stranded RNA, as well as a RNA binding protein named DGCR8/Pasha (15). In the cytoplasm, another RNase enzyme (called Dicer) activity leads to generation of the mature miRNAs. Functionally, Dicer cleaves the terminal loop of pri-miRNA and produces double stranded 19-22 nucleotide miRNAs (16). Usually only one strand of the mature miRNAs, known as the guide strand, enters into the micro-ribonucleoprotein complex and creates a micro-RNA-induced silencing complex (miRISC), where the sequence of this strand determine binding region at the target mRNA (17,18). Since only one of the double strands has the ability to play the guidance role for directing the RISC to the 3'UTR region of the target mRNA, the second strand is deleted. RISC binding miRNAs pair to the 3'UTR region of the target mRNA homologous and control gene expression by inhibiting the cleavage or translation of mRNA targets (19,20). About one-third of the human genome is considered as potential regulatory targets by the several hundred miRNAs encoded in the genome. Such regulation happens post-transcriptionally and comprises the interaction of miRNA with the mRNA target site (Fig.1). 

## Mammalian target of rapamycin signaling pathway

The mammalian target of rapamycin (mTOR) signaling pathway integrates inputs from both intracellular and extracellular signals to regulate different cellular processes including proliferation, growth, survival, motility, autophagy, protein synthesis and metabolism. mTOR is a downstream effector of the PI3K/ AKT pathway and consists of two biochemically distinct complexes, including mTORC1 and mTORC2. mTORC1 promotes anabolism, such as cell cycle progression, and inhibits catabolism by blocking autophagy. Signaling of this complex contributes to tumorigenesis through its major downstream targets and key regulators, namely 4E-BPs. It has been demonstrated that mTORC2 regulates cell survival, proliferation and metabolism. Furthermore, mTORC2 is responsible for phosphorylation and activating AKT, which may drive tumorigenesis (21,22). Recent studies have revealed different roles for mTOR in modulating lifespan, considering two processes that mTOR regulates, including protein synthesis and autophagy (23). Another study reported that mTOR is increased in association with BMAL1 deficiency, a transcription factor and core component of an internal time-keeping system called circadian clock. This event eventually contributes to premature aging and reduced lifespan (24). Wide-ranging researches have indicated that miRNAs-based regulation of the mTOR pathway plays a key role in cancer progression, and this pathway is a promising target by miRNAs for novel anticancer therapies (21). Jin et al. (25) in a study on the animal model identified a panel of 63 miRNAs during dermal wound healing, including miRNA-99 family (miRNA-99a, miRNA-99b, and miRNA-100). They demonstrated that miRNA-99 family members regulate AKT/mTOR signaling by targeting several genes such as IGF1R. Grundmann et al. (26) screened miRNAs involved in adaptive blood vessel growth following arterial occlusion. They showed that inhibition of miRNA-100 could be a novel approach for the modulation of mTOR-dependent processes, such as blood vessel growth. A growing body of evidences suggests that miRNAs may play a crucial role in cancer therapy and diagnosis, which mostly performed through the mTOR signaling pathway (Table 1). 

## miRNAs link with cellular senescence, ageing and cancer

Well understanding of the cancer molecular mechanisms pathogenesis and active targeted therapies are necessary to improve patient treatment outcomes. miRNAs act as key components in cancer progression and as the potential therapeutic agents or targets. Numerous studies have suggested that miRNAs inhibit tumor proliferation and promote cellular senescence or ageing, but its function has yet to be elucidated. Other studies reported that miRNAs repress global translation, cell proliferation and initiates premature senescence (Table 2). 

## miRNAs, ageing and epigenetics

**Fig.2:**
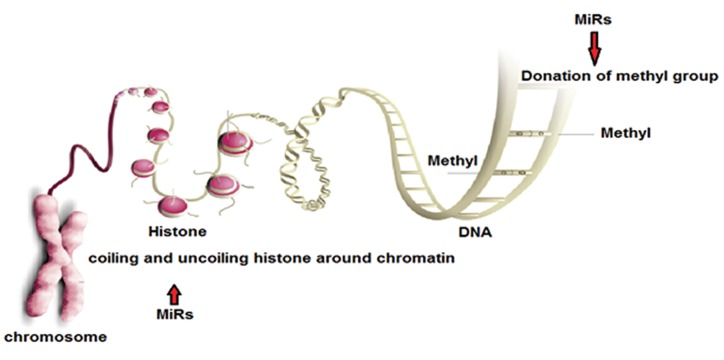
Two prominent mechanisms involved in regulation of gene expressions by miRs (miRNAs).

Ageing is a potent predictor of survival rate in cancers, while the biological mechanisms for the variation in clinical outcome are mostly unidentified. Determining genes and pathways, which are responsible for age-related survival changes, could facilitate the chance of novel therapeutic establishments. Bozdag et al. (38) have integrated various molecular and genetic methods to determine age-specific signatures at the genetic and epigenetic levels in glioblastoma multiforme. Ageing of higher organisms are regulated by the epigenetic variation over time. Some epigenetic changes do not follow any determined roles, suggesting that might be the outcome of epigenetics error accumulations. Thus, when this process takes place in adult stem cells, it could play an important role in ageing, through some unknown molecular mechanisms (39). Many researches have discussed various mechanism that miRNA could affect DNA methylation as an epigenetic change contributing to ageing and cancer (Table 3). Two main epigenetics components are DNA methylation, methyl marks add to a certain bases of a gene, and histone modification, combination of various molecules attached to the tails of histone proteins. Functionally, miRNAs could regulate gene expressions through two prominent mechanisms, including donation of the methyl group (40) and chromatin coiling/uncoiling (Fig.2) (41). Wakabayashi et al. (42) revealed that there is likely a cross-talk between miRNAs and epigenetic regulators, modulating neurogenesis in the adult mammalian brain. 

**Table 1:** Linkage between miRNA and mTOR signaling pathway


Authors	Cell line(s)	Type of disease	Type of miRNA	Target^*^	Finding/Suggestion for miRNA

Zheng et al. (27)	SGC-7901 cell	Gastric cancer	miRNA-18a	Vacuolar protein sorting- associated protein 13D	Possible therapeutic strategy against malignancy
Wan et al. (28)	CNE and HeLa cells	Hypoxia- induced autophagy	miRNA-155	Kinesin-like protein KIF1B Nuclear factor 1 A-type	A key regulator of autophagy via dysregulation of mTOR pathway
Shen and Houghton (29)	CB17SC SCID mice	Childhood sarcoma	miRNA-18a	Vacuolar protein sorting- associated protein 13D	Oncogenic growth signals may promote tumorigenesis by dampening the ATM checkpoint
			miRNA-421	Calmodulin-binding transcription activator 1 Arginine-glutamic acid dipeptide repeats protein	
Zhong et al. (30)	Colorectal carcinoma cell	Colorectal carcinoma	miRNA-30a	FUS-interacting serine- arginine-rich protein 1	A potential therapeutic target to block CRC metastasis
			miRNA-30b	Kinesin-like protein KIF1B MARCKS-related protein	
Wang et al. (31)	Mouse adult pancreatic islets	Diabetes	miRNA-7	Kinesin-like protein KIF1B FKBP12-rapamycin complex-associated protein	As a therapeutic target for diabetes
Li et al. (32)	MG63 cells	Osteosarcoma	miRNA-223	AR DNA-binding protein 43 Msx2-interacting protein FUS-interacting serine- arginine-rich protein 1	Could be used in anticancer therapies in osteosarcoma
Cui et al. (33)	Renal cell Carcinoma cell	Renal cell carcinoma	miRNA-99a	Uncharacterized protein C1orf34 plasticity related gene 1	May offer an attractive new target for diagnostic and therapeutic interventions
Iwaya et al. (34)	HT29 and CaR-1 cell	Colorectal carcinoma	miRNA-144	PR domain zinc finger protein 2 Msx2-interacting protein	A meaningful prognostic marker
Gebeshuber and Martinez (35)	Breast cancer cell	Breast cancer	miRNA-100	TAR DNA-binding protein 43 Uncharacterized protein C1 or f34	A potential target for therapeutic approaches


*; Predicted by target scan and mTOR; Mammalian target of rapamycin.

**Table 2:** miRNA linking cellular senescence, ageing and cancer


Authors	Type of disease	Type of miRNA	Target^*^	Finding/Suggestion for miRNA

Liu et al. (36)	Ovarian carcinoma	miRNA-506	Calmodulin-binding transcription activator 1 Msx2-interacting protein	Inhibits proliferation while promotes senescence
Mazan-Mamczarz et al. (37)	Diffuse large B cell lymphoma	miRNA-520c-3p	Kinesin-like protein KIF1B Nuclear factor 1 A-type	A key regulator of autophagy via dysregulation of MTOR pathway.
Shen and Houghton (29)	Childhood sarcoma	miRNA-18a	Vacuolar protein sorting-associated protein 13D	Oncogenic growth signals may promote tumorigenesis by dampening the ATM checkpoint
		miRNA-421	Calmodulin-binding transcription activator 1 Arginine-glutamic acid dipeptide repeats protein	
Zhong et al. (30)	Colorectal carcinoma	miRNA-30a	FUS-interacting serine- arginine-rich protein 1	A potential therapeutic target to block CRC metastasis
		miRNA-30b	Kinesin-like protein KIF1B MARCKS-related protein	
Wang et al. (31)	Diabetes	miRNA-7	Kinesin-like protein KIF1B FKBP12- rapamycin complex- associated protein	As a therapeutic target for diabetes
Li et al. (32)	Osteosarcoma	miRNA-223	AR DNA-binding protein 43 Msx2-interacting protein FUS-interacting serine- arginine-rich protein 1	Could be used in anticancer therapies in osteosarcoma
Cui et al. (33)	Renal cell carcinoma	miRNA-99a	Uncharacterized protein C1 or f34 plasticity related gene 1	May offer an attractive new target for diagnostic and therapeutic intervention
Iwaya et al. (34)	Colorectal carcinoma	miRNA-144	PR domain zinc finger protein 2 Msx2-interacting protein	A meaningful prognostic marker
Gebeshuber and Martinez (35)	Breast cancer	miRNA-100	TAR DNA-binding protein 43 Uncharacterized protein C1 or f34	A potential target for therapeutic approaches


*; Predicted by target scan and mTOR; Mammalian target of rapamycin.

**Table 3:** miRNA affects DNA methylation as an epigenetic change contributing to ageing and cancer


Authors	Type of disease	Type of miRNA	Target^*^	Finding/Suggestion for miRNA

Ng et al. (43)	Acute promyelocytic leukaemia	miRNA-34a	Kelch-like protein 17 (Actinfilin) Calmodulin-binding transcription activator 1	Methylation of miRNA-34b/c may contribute to APL leukaemogenesis
		miRNA-34b	Kelch-like protein 17 (Actinfilin) Tumor protein p73 (p53-related protein)	
		miRNA-34c	Kelch-like protein 17 (Actinfilin) PR domain zinc finger protein 16	
Xie et al. (44)	Hepatocellular carcinoma cancer	miRNA-34a	Kelch-like protein 17 (Actinfilin) Calmodulin-binding transcription activator 1	DNA methylation might be involved in the inactivation of miRNA-34b in HCC
		miRNA-34b	Kelch-like protein 17 (Actinfilin) Tumor protein p73 (p53-related protein)	
		miRNA-34c	Kelch-like protein 17 (Actinfilin) PR domain zinc finger protein 16	
Ko et al. (3)	Acute myeloid leukemia	miRNA-let-7a	Basement membrane-specific heparan sulfate proteoglycan core protein precursor	let-7a-3 methylation is a positive prognosticator for AML patients
Huang et al. (45)	Endometrial cancers	miRNA-203	Msx2-interacting protein Macoilin AT-rich interactive domain-containing protein 1A	miRNA-203 methylation Level might represent a marker for the patients with endometrioid cancers
Verhoef et al.(46)	Cervical intraepithelial neoplasia grade 2 (CIN2)	miRNA-124a-2	Arginine-glutamic acid dipeptide repeats protein retinoblastoma-associated factor 600	DNA methylation of miRNA-124-2 on HPV-test-positive self-samples is non-inferior to cytology triage in the detection of CIN2
Li et al. (47)	Non-small cell lung cancer cells (NSCLC)	miRNA-503	Protein kinase C zeta type Vacuolar protein sorting-associated protein 13D	Epigenetic silencing of microRNA-503 regulates FANCA** expression in non-small lung cancer cell
Ben Gacem et al. (48)	Breast cancer	miRNA-124a-1 miRNA-124a-2 miRNA-124a-3	Arginine-glutamic acid dipeptide repeats protein retinoblastoma-associated factor 600	DNA methylation of miRNA-124a-1, miRNA-124a-2 and miR-124a-3 in breast cancer play a role in tumor growth and aggressiveness
Wang et al. (49)	Chronic lymphocytic leukemia (CLL)	miRNA-9-3	Nuclear inhibitor of protein phosphatase 1 Eyes absent homolog 3 Zinc finger MYM-type protein 6	miRNA-9-3 is a tumor suppressor miRNA frequently methylated, and hence is silenced in CLL
Yamada et al. (50)	Acute lymphoblastic leukaemia	miRNA-128a	Integrator complex subunit 11	Induction of miRNA128a expression by DNA demethylation is a novel mechanism of resistance to Fas-mediated apoptosis
Nadal et al. (51)	Lung adenocarcinoma	miRNA-34b	Kelch-like protein 17 (Actinfilin) Tumor protein p73 (p53-related protein)	Epigenetic inactivation of miRNA-34b/c by DNA methylation has independent prognostic value in patients with early-stage lung adenocarcinoma
		miRNA-34c	Kelch-like protein 17 (Actinfilin) PR domain zinc finger protein 16	
Xing et al. (52)	Hepatocellular carcinoma	miRNA-122	Arginine-glutamic acid dipeptide repeats protein Msx2-interacting protein	DNA Methylation of miRNA-122 might be involved in the development of hepatocellular carcinoma
Zhang et al. (53)	Nickel-induced cancer	miRNA-203	Msx2-interacting protein Macoilin AT-rich interactive domain-containing protein 1A	DNA methylationassociated silencing of tumor suppressor miRNAs contributes to the development of Nickelinduced cancer
Zhang et al. (54)	Colorectal cancer (CRC)	miRNA-126	D site-binding protein Protein LAP2 Uncharacterized protein C10orf6	DNA methylation-induced silencing of miRNA-126 contributes to tumor invasion and angiogenesis in CRC


*; Predicted by target scan and **; Fanconi anemia complementation group A protein.

## miRNA therapeutic applications

miRNA detection has opened a new window in our current perception of the gene expression regulation. Similar to protein-coding genes, several investigations have been performing to determine the expression level of these small RNAs *in vitro* or *in vivo*. Hence, miRNAs might undergo gain of function (GOF) or loss of function (LOF). This event could play an important role in various diseases-like protein-coding genes. Different mechanisms including genomic rearrangement, point mutation, and altering the pattern of promoter region methylation could be involved in regulation of miRNA expressions. Besides, this type of RNA plays an important role in expression and regulation of signaling pathways. It is necessary to evaluate the relationship between aberrant miRNA, like miRNA-128 and miRNA-30, expression levels and notch signaling in glioma and angiogenesis, respectively (55).

Several studies have shown that expression or inhibition of miRNAs can change the pattern of tumorigenesis or cancer progression (56-59). It has been demonstrated that expression of several miRNAs (e.g. miRNA-17, miRNA-155) might have oncogenic properties, while the others (e.g. miRNA-34, miRNA-16 and let-7) function as tumor suppressor (60, 61). Here, we suggest that oncogenic or inhibitory effect of miRNAs could raise a distinctive point to compare the normal cells with different types of cancer. Thus, analysis of miRNA expressions, as a molecular bio-marker, could help diagnose the patient’s disorder stage. For example, over-expression of miRNA-155 and down-regulation of let-7 indicated low survival chance in the patients with lung cancer (62, 63). Curiously, the expression pattern of some miRNAs is associated with different stages of tumorigenesis or metastasis, proposing their potential benefit to use as bio-markers (63). Generally, miRNAs can prevent cancer progression through inhibiting the other oncogenic miRNAs, by degrading mRNA through binding with miRNA, inducing tumor suppressor miRNAs or down-regulating the expression level of other miRNAs by regulating epigenetic factors, such as methylation of the gene promoter (64, 65). In contrast, anti-sense oligonucleotides paired with miRNAs can reduce the expression of these small RNAs (66).

## Discussion

Currently, there are several types of synthetically made miRNA. Antagomir is an example of this type of artificially made miRNAs. These RNA molecules are designed to inhibit miRNAs. The precise mechanism that anatgomir could inhibit miRNAs is not clear yet, although this mechanism might possibly be performed where these molecules could irreversibly bind to miRNAs. miRNA-based therapeutics could be applied through two approaches; in the first approach, miRNA antagonist applications (e.g. antagomir, anti-miRNA and LNA) contribute through GOF. In the second strategy, using inhibitory miRNAs (e.g. tumor inhibitors) could lead to LOF, compensating lack of natural intracellular miRNAs function. This strategy is similar to transferring protein-coding genes into cells during gene therapy, with even less limitations due to the small size of transferred DNA. Thus, it can easily be transferred into the cells using chemicals without any vector, like inhibitory RNA delivery. In addition, the nature of miRNA function is the other benefit which is mostly influenced by multiple oncogenic paths. Delivery of tumor suppressor miRNAs is mainly done by viral vectors. Another inhibitory transmission approaches, direct miRNAs to the target organ using plasmids, transposons and cationicliposome, as monoclonal antibodies embedded on their surface, epigenetic modifying drugs such as DNA methyltransferase inhibitors (including 5-aza-2'deoxycytidine), histonedeacetylase inhibitors (including 4-phenylbutyric acid) increase the expression of miRNA by reducing DNA methylation and increasing histoneacetylation level, as well as inhibiting cell proliferation through reversing the tumor suppressor effect of miRNA (67). Thus far, several miRNA inhibitors have been introduced to preclinical studies in animal models, one of the most prominent of which is let-7 (68,70). The expression of this miRNA inhibitor is reduced in some cancers, leading to inhibitory effects on the RAS protein family. Furthermore, reduction or loss of activity of this miRNA inhibitor leads to increase in the expression of these proto-oncogenes (71). 

These miRNAs also affect other targets such as MYC, cyclin D and HMG2A, indicating the importance of such miRNAs in controlling several pathways related to cancer (72). miRNA-34a, as a target of P53, is another small RNA that prevents the growth of cancer cells by controlling the cell cycle (73). In addition to these direct applications of miRNA in cancer therapy, adjuvant administrations have been discovered for these RNAs. For example, it has been shown that transferring and expressing miRNA-302 in breast cancer cells enhances the sensitivity of these cells to radiotherapy (74). However, the important point, regarding miRNAs replacement therapy, is the risk of cellular toxicity. As demonstrated, miRNAs are required to be proceeded by the RISC. Transferring high amounts of miRNAs to the cells can, in contrast, decrease or omit the other natural miRNAs processing by this complex, which could negatively affect the cell survival. Recently in the field of cancer and ageing, scientists claimed that uric acid can be used to regulate ROS, preventing cancer and ageing. These findings highlight the role of miRNA-based inhibiting the SLC2A9 antioxidant pathway in cancer as a novel approach to kill malignant cells while a patient is fighting with the cancer (75). 

## Conclusion

The aforementioned evidences illustrate the complexity and the dynamic nature of miRNAbased linkage of ageing and cancer at genomics and epigenetics levels that might be crucial for the understanding of the age-related cancer mechanisms and ageing, in general. 
